# Understanding the impact of the COVID-19 pandemic on U.S. older adults: self-reported pandemic-related concerns and consequences in a cross-sectional survey study

**DOI:** 10.3389/fpsyg.2023.1203473

**Published:** 2023-11-16

**Authors:** Nichole Sams, Doyanne Darnell, Dylan Fisher, Ryan Allred, Kathy Huyhn, Brittany A. Mosser, Patricia A. Areán

**Affiliations:** ^1^Department of Psychiatry and Behavioral Sciences, University of Washington, Seattle, WA, United States; ^2^CREATIV Lab, University of Washington, Seattle, WA, United States; ^3^ALACRITY Center, University of Washington, Seattle, WA, United States

**Keywords:** COVID-19, mixed-method, older adults, qualitative, mental health

## Abstract

**Background and objectives:**

The purpose of this study was to explore COVID-19 pandemic-related concerns among a racially and ethnically representative sample of older adults in the U.S.

**Research design and methods:**

Participants were 501 English-speaking adults 60 years and older recruited online nationally across the U.S. from Amazon Mechanical Turk (mTurk) and Prolific Research Platforms during June of 2020. Data comes from a larger cross-sectional survey. We content analyzed open-ended responses about pandemic-related concerns and observed responses to a checklist of items created by the research team to assess for specific physical, social, and financial consequences experienced due to the pandemic.

**Results:**

A majority of the sample (92%) reported at least one pandemic-related concern, with the highest percentage expressing concerns coded as Concern for Others (28%), Physical Health (27%), Socializing (24%), Finance (15%) and Socio-Political-Economic (14%). Participants reported high concern severity (*M* = 4.03, *SD* = 1.04) about their concerns mentioned in response to the open-ended concerns question. When prompted with a checklist of items, participants frequently endorsed disruption in social activities as a consequence of the pandemic (83%), disruptions that could impact physical health (45%), and concern over finances as a consequence of the pandemic (41%).

**Discussion and implications:**

Older adults most frequently mentioned concerns about the well-being and behavior of others, one’s own physical health, and the impacts of the pandemic and social distancing policies on social activities. Findings align with the Socioemotional Selectivity Theory and point to the importance of supporting older adults to maintain meaningful social engagement under conditions of a pandemic and social distancing policies.

## 1. Introduction

On March 11th, 2020 the World Health Organization (WHO) declared the Coronavirus 2019 (COVID-19) a pandemic. To limit the spread of the virus in the U.S., governments required “social distancing” and shut down non-essential businesses, while retaining only essential business operation with precautions (e.g., wearing a mask) (State of California Executive Department, 2020; State of New York Executive Chamber, 2020). Older adults are considered to be a group vulnerable to the negative impacts of the pandemic given that they are more likely to suffer death, medical complications, and negative consequences related to social isolation than other age groups. Although these known vulnerabilities may be a concern among older adults themselves, a richer understanding of the psychosocial impact of the pandemic on older adults may be gained by inquiring in an open-ended fashion about the nature of their concerns related to the pandemic. Few studies have made such an inquiry; therefore, the present study aims to fill this gap in the literature on the experience of older adults during the early phase (3 months out from the WHO declaration) into the COVID-19 pandemic.

### 1.1. The impact of the COVID-19 pandemic on older adults

Research on the impact of the COVID-19 pandemic on older adults point to a variety of negative consequences for their well-being. For instance, in 2022, the Centers for Disease Control (CDC) reported that 74% of COVID-19 deaths were among persons 65 and older (Tejada-Vera and Kramarow, 2022), and studies indicate older adults experienced challenges accessing health services during the pandemic (Scott et al., 2021). Active engagement and access to healthy lifestyle and healthcare are essential components of healthy aging (Rowe and Kahn, 1997) and was made difficult by the pandemic. Pre-pandemic, 11,000 senior centers across the U.S. served over 1 million older adults daily, and many of these services were interrupted, cancelled, or altered (NCOA, 2020; Administration for Community Living, 2020a). In ages 55 and older, unemployment went up from February (3%) to April (14%) (Bureau of Labor Statistics, 2020). A noted public health concern was the potential for social distancing policies to worsen social isolation that is often already heightened within the older adult population (Macleod et al., 2021).

#### 1.1.1. Impact on psychological well-being

It is well-documented that the stress of adverse community events such as natural or technological disasters, terrorist attacks, mass violence and pandemics cause psychological distress and impact psychological functioning (Hobfoll et al., 1995; Norris et al., 1999). However, longitudinal research on the psychological impact of COVID-19 suggests that although the pandemic was associated with early increases in distress, over time this distress decreased to pre-pandemic levels (Daly and Robinson, 2021; Robinson et al., 2022) and older versus younger age was associated with less distress during the pandemic and better emotional well-being (Carstensen et al., 2020; Wilson et al., 2021; Best et al., 2023). There is some indication, however, that there may be more subtle fluctuations of psychological distress when age groups are further broken down. For instance, results of two longitudinal studies suggest the older-older adults (e.g., age 85) were more negatively impacted psychologically by COVID-19 over the long-term but less negatively impacted in the short-term than younger-older adults (e.g., age 65; Segerstrom et al., 2023).

#### 1.1.2. Pandemic-related concerns of older adults

Survey studies with older adults indicate older adults commonly experienced impacts of the pandemic on areas of life having to do with health behaviors (e.g., increased alcohol use, decreased physical activity), reduced access to health care, reduced engagement in social activities, and financial concerns (Scott et al., 2021; Robinson et al., 2022). Fewer survey studies have qualitatively explored older adults’ experiences with and concerns about the pandemic. A cross-sectional survey of 825 U.S.-residing predominantly White (97%), female (80%), Midwest-dwelling (47%), adults ages 60 and older inquired about stressors and joys amidst the pandemic (Whitehead and Torossian, 2020). Participants were recruited through university email listservs and social media posts from March 22–23, 2020. Participants were asked, “What are you finding most challenging or stressful today?*”* The researchers’ coding scheme included stressors that could be considered both associated with the pandemic and not associated with the pandemic, or more general. The most common were confinement/restriction (13%), concern for others (12%), isolation and loneliness (12%), and uncertainty of the future (9%). Additional themes that appeared included shopping (7%), government response (7%), the news (7%), economy/finances (6%), getting the virus (4%), and how other people were responding to the pandemic (4%).

Another cross-sectional online survey enrolled 235 older adults from Oregon ages 51–95, predominantly non-Hispanic White (92%) and female (74%) as part of the Life Registry study from April 28th – May 4th, 2020 (Igarashi et al., 2022). The survey asked participants the open-ended question “During the past week, what was the most difficult thing for you about the COVID-19 Situation?” The researchers’ coding scheme included 9 total themes including difficulties with every day protective activities (EPAs) and consequences (45%), psychological distress (25%), worry about personal finances (1%), struggles with interpersonal connections (30%), concern for close others (7%), cultural divide (20%), concern for society (9%), reaction to COVID-19 cases and deaths (5%) and concern for community others (5%).

Older adults in a larger sample of 1,272 adults aged 64 and older (91% White and 61% female) in New Jersey were asked in May of 2020 “Take a moment to reflect on your overall experiences with COVID-19 since mid-march of 2020. Of all the changes taking place, what has been most difficult for you?” (Heid et al., 2021). Responses were content analyzed. The most frequently mentioned challenges involved social relationships, such as missing social interactions with friends, family, coworkers or others (42%) and activity restrictions, such as restriction on leisure, shopping, church, etc. (31%). Much less frequently mentioned were categories identified as psychological stressors, such as worries or concerns related to getting COVID-19 (3%) and the well-being of other people (5%) or stressors related to health, finances, global environment, death, and home care.

### 1.2. The present study

Qualitative inquiry into self-reported concerns related to the COVID-19 pandemic within the context of survey research can provide both breadth and depth to understanding the psychological impact of the pandemic on older adults. The present study adds to the growing body of literature on qualitative inquiry into the impact of the pandemic on U.S. older adults by uniquely recruiting a nationally representative sample with regards to race and ethnicity, recruiting older adults via online platforms, and exploring this data alongside quantitative observation of the impact of the pandemic on activities of older adults. Specifically, we aimed to explore the types of concerns that U.S.-based older adults had related to the COVID-19 pandemic based on their responses to an open-ended survey question. Additionally, we utilized responses to quantitative items on the larger survey inquiring about whether older adults experienced a variety of social, health, and economic consequences due to the pandemic to better understand the impact of the pandemic on their lives.

## 2. Methods

### 2.1. Design and participants

Data for this study come from a larger national, cross-sectional survey study that included both quantitative and open-ended qualitative questions. Participants were 501 English-speaking older adults (60+ years old) living in, and graduated from high school, in the U.S. Participants were recruited through two online platforms: Prolific Workforce and Amazon Mechanical Turk (MTurk), and therefore had access to a device connected to the internet and the ability to take online surveys (Buhrmester et al., 2011; Palan and Schitter, 2018). We selected these platforms because they permit users to efficiently complete surveys and other tasks in exchange for compensation and based on previous research highlighting the benefits of such platforms to efficiently collect data with populations with diverse backgrounds (Peer et al., 2017; Palan and Schitter, 2018). The study recruited a racially representative sample as defined by the U.S. census data from 2010: White (75%), Black or African American (14%), American Indian or Alaska Native (2%), Asian (6%), Native Hawaiian/Pacific Islander (<1%) two or more races (3%; U.S. Census Bureau, 2010). The research was approved by the University of Washington’s institutional review board.

### 2.2. Procedures

Participants were solicited between June 16, 2020 and June 25, 2020. The advertisement read “We are conducting an academic survey about older adults’ experiences during COVID-19 related to social isolation. We will be asking you questions about your mood, activities, and well-being and how those have changed.” Participants provided informed consent online before completing the anonymous survey, after answering questions to confirm their understanding of the consent details and being given an opportunity to ask the study team questions. Participants were then provided a link to a 20–30 min online survey through REDCap (Harris et al., 2009). The REDCap installation hosted at UW collected and stored the data securely. Participants were remunerated $5 via their MTurk or Prolific account.

### 2.3. Measures and variables

#### 2.3.1. Demographic questions

Participants self-reported demographics, which included age, gender identity (Female, Male, Self-Describe, No Answer), racial and ethnic identity (American Indian/Alaska Native, Asian, Black/African-American, White/Caucasian, Hispanic/Latinx and/or Multi-racial), whether they live alone versus with others, marital status (Divorced, Married or Partnered, Never Married, Separated, Widowed), whether they are financially able to “make ends meet” versus “have just enough to get by” versus “are comfortable,” total income over the previous year, whether they were insured by Medicaid or not, and highest education level (in years).

#### 2.3.2. Pandemic related concerns and concern severity

Participants responded to an open-ended self-report question, “Of all the things that have happened to you since the COVID-19 pandemic started, what concerns you the most? Please provide as much detail and description about your concern.” Additionally, an item assessed how concerning this is to participants on a scale of 1 (not at all) to 5 (very much). These questions were adapted from previous research assessing patient concerns following hospitalization for a serious injury (Zatzick et al., 2001).

#### 2.3.3. Pandemic-related consequences checklist

The research team developed a checklist of 10 items to assess potential consequences of the COVID-19 pandemic that could negatively impact older adults’ physical, social, or financial well-being. Participants indicated whether each item had been a consequence for them due to COVID-19: (1) Disruptions in needed medical care, (2) concern over finances, (3) not attending social events or group activities at your residence (shared meals, movie nights, etc.), (4) not attending social events or group activities in the greater community (movie theatres, book clubs, game nights, etc.), (5) not going outside, (6) not running usual errands, (7) not seeing family, (8) not getting physical exercise, (9) difficulty acquiring groceries or other essentials, or (10) other consequences not listed. Participants were asked these checklist items immediately prior to being asked the open-ended pandemic-related concerns question.

### 2.4. Plan of analysis

#### 2.4.1. Descriptive statistics

We observed descriptive statistics for participant characteristics, the pandemic-related consequences checklist items, and types of concerns mentioned by participants in the open-ended pandemic-related concerns question based on the content analysis of participant responses to this question (described below). Descriptive statistics included frequencies for categorical variables and the frequencies of types of concerns as well as measures of central tendency (e.g., mean, standard deviation) for continuous variables.

#### 2.4.2. Content analysis of pandemic-related concerns

Open-ended responses from the pandemic-related concerns question were content analyzed (Neuendorf, 2017) to characterize the types of concerns reported and their frequency across participants. We developed a coding scheme based on *a priori* codes selected from the coding scheme used in previous research with injured trauma patients (Zatzick et al., 2001) as well as codes identified emergently following a review of participant responses. Participant responses were the unit of analysis.

Authors DD and NS reviewed each participant response and developed the initial coding scheme and coding manual. All 501 participants gave a valid response to be reviewed for a code. NS trained three additional coders to apply the coding scheme with 10 randomly-selected responses. Coders had or were completing a bachelor’s degree in a social science field and were research staff at the University of Washington. Two coders identified as female and two as male. These four coders piloted the coding scheme by first independently coding 40 randomly selected responses followed by consensus-coding those same responses to iterate on the coding scheme. These four coders then independently coded 50 randomly selected responses for a final estimate of inter-rater reliability and consensus coded these responses. The remaining 401 responses were randomly assigned and independently coded by each coder.

## 3. Results

### 3.1. Participant demographics

Participants were primarily female (*n* = 324, 65%), college educated (*M* = 15.26 years), Non-Hispanic European American (*n* = 377, 75%) and living with others (*n* = 346, 69%; see Table 1). Half the sample was married or partnered (*n* = 260, 52%). Less than half the sample indicated they were financially comfortable (*n* = 187, 37%), while 48% (*n* = 240) endorsed having just enough to get along, and 10% (*n* = 51) said they could not make ends meet.

**Table 1:** Participant demographics.

	Mean	Std Dev.
Age	65.5	4.59
Education (in years)	15.26	2.6
Gender identity	*n*	%
Female	324	64.7
Male	165	32.9
Self-describe	0	0.0
No answer	12	2.4
Racial and ethnic identity		
American Indian/Alaska Native	2	0.4
Asian	17	3.4
Black/African-American	56	11.2
White/Caucasian	377	75.2
Hispanic/Latinx	27	5.4
Multi-Racial	22	4.4
Living situation		
Alone	154	30.7
With others	346	69.1
Marital status		
Divorced	127	25.3
Married or partnered	260	51.9
Never married	72	14.4
Separated	12	2.4
Widowed	30	6.0
Perceived financial comfort		
Are comfortable	187	37.3
Have just enough to get along	240	47.9
Cannot make ends meet	51	10.2
Income categories		
<9,000	22	4.4
9,000 – 12,999	24	4.8
13,000 – 27,999	117	23.4
28,000 – 49,999	128	25.5
50,000 – 74,999	100	20.0
75,000 – 99,999	60	12.0
100,000<	49	9.8

### 3.2. Pandemic-related concerns and concern severity

One hundred of 501 participant responses were consensus coded as part of training coders and estimating inter-rater reliability, with 401 responses then independently coded. The final coding scheme included 11 categories of concerns (see Table 2). For each category, coders determined whether the category was mentioned or not mentioned. If coders were unsure whether the code was mentioned, they were instructed to code the category as not mentioned for that response. The codes were not mutually exclusive, so one participant response may have more than one code mentioned (or no codes mentioned at all). Data was entered into SPSS (IBM Corp., 2019) for analysis. Interrater reliability estimates using Fleiss’s Kappa could not be calculated for low-frequency codes since there were insufficient observations from which to estimate; these included Access to Social Services, Access to Hygienic or Household Supplies and Spiritual. Estimates for all other codes ranged from 0.8–1.0 (substantial to almost perfect agreement), except for Psychological Health and Mental Wellness, which were 0.64.

**Table 2:** Qualitative coding definitions for types of concerns reported in the open-ended pandemic-related concerns question.

Physical Health	Expresses any concern about the participant’s physical health, including contracting COVID-19, worsening conditions due to COVD-19, or concerns related to pre-existing conditions or obtaining healthcare for physical conditions in general *“I am most concerned about catching COVID through my daily commute or at work. One person where I work has already died from COVID. I do not know how many people at work are infected”* *“I am now both housing and income insecure//plus I’m 62, a former smoker and overweight so I’m Hi Risk X3 // the wealthy will be fine, me maybe not so much”* *“I am afraid that if I should have an emergency my family would not be able to come to take care of me or if I would be able to keep my regularly scheduled medical appointments”*
Psychological Health and Mental Wellness	Expresses concern about the participant’s mental wellness or psychological health. This includes emotional impacts of the pandemic, psychiatric diagnosis or general emotional distress e.g.: depression, loneliness, sadness, not wanting to live etc. Should capture when the participant mentions their mental wellness being impacted or concern of loss of access to activities and tools they use to stay mentally well. *“The constant worry about sanitizing, washing hands, wiping things down, staying away from others. It is very draining emotionally for me to always be hypervigilant.”* *“I am most concerned about my emotional state because the rising number of cases is exacerbating my anxiety.”* *“it makes me angry and I’m concerned about my mental and physical health when I live in a condition of worry and anger.”* *“My mental state is a mess. I am severely depressed, can barely eat, am drinking too much for relief, wake up afraid and stressed.”*
Work/Finance/Employment	Expresses a concern about one’s personal work, employment or finances. This could include concerns about losing jobs, cutting back of hours, using savings etc. This code applies to participants’ own finances, not a mention of the general economy, which is covered in the Social-Political-Economic code. *“probably the financial concerns are the greatest. We are trying to fit within a budget that allows us to stay in our rented apartment and still have money for food, gas, medical,* etc. *We are on a payment plan for our debts, but they will never be paid off now. We doing pretty well financially before, but it is all up in the air now. We are on a fixed income, so are really worried about price of food and medical going up so much that we cannot afford medication or food, or will have to leave our apartment. We had to give up our smart phones so that provided a little more isolation.”* *“I will likely retire a bit earlier due to ongoing risk for Covid-19. My job is held for me at the same rate of pay 3/18 to 7/30 but I expect the expectations and responsibilities to be different, and safety only partly addressed”*
Access to Social Services	Expressing concern about personal lack of access to social services. Does not include access to medical, mental health or addiction services as these are included in code categories Physical Health and Psychology Health and Mental Wellness. *“My other concern is I hope the US Postal Service will not get changed in any way because it is a convenient source for the older people when they can mail their letters from the mailbox.”*
Access to Food	Expresses a concern about not having access to food, such as not being able to partake in meals delivery services or being able to cook or obtain food for self. *“The grocery stores running out of toiletries and meats as well as dry goods such as pasta and cereals. Having to go from store to store is very stressful and time consuming. It’s a very exhausting experience.”*
Access to Hygienic or Household Supplies	Expresses a concern about not having access to hygienic or household supplies or difficulty getting them. These might include things like toilet paper, hand sanitizer, masks or other supplies that might be impacted because of the pandemic. “*Getting household and personal essentials is still hard to find and get.”* *“breakdown of supply lines. Now there is a shortage of appliances, as factories all over the world have been shut down”*
Mask Wearing	Expressing any mention of mask wearing, aside from having access to mask supplies. *“I am concerned that I live in a community and state where people do not take the pandemic seriously, are not following the recommended guidelines for safety and even in some cases being angry at those that do. This concerns me because it isolates me more as I’m not going out if people aren’t wearing masks and I’m afraid that the pandemic will come back even worse.”* *The*” *I’m an American and I do not have to wear a mask if I do not want to” mentality is absolutely nuts. I go to the store about once every 8 or 9 days. I’m always surprised to see how many people are going in without masks and with 2 or 3 children in tow.*
Socializing	Expresses concerns about not being able to be around others and or connect with others, such as spending time with relatives, friends, and/or doing activities that involve other people. For example, this could be family or community events, whether the participant is directly interacting with other people present. This can also include concerns about the pandemics’ impact on typical connection with others, such having to utilize technology to connect remotely instead of in-person. “*I really miss the hugs from my family and friends. I’ve stepped back from family so they would not touch me and it’s heartbreaking to do so with my youngest 4-year-old great niece.”*
Socio-Political- Economic	Expresses a concern about social, political or economic issues that are related to the pandemic or happening contemporaneously. Economic issues are about community or society at large, not specific to the participant’s economic well-being, which is assessed with code Work/Finance/Employment. This includes concerns about the government response to the pandemic, local or federal, civil/human rights issues and protests, or mentions a concern specific to a particular sociopolitical identity group *“What concerns me the most is the way so many Americans are thinking about COVID-19. Trump sort of made it seem insignificant and so many people believed him. I’m not talking about any other person or any other Republican, just Trump.”* *“childish fears of others,* i.e.*, Biden and his mask attack months after the virus is gone.”* *“I am also concerned about a collapse of the economy.”*
Spiritual	Participant mentions a concern regarding their spirituality or spiritual/religious practices. Examples might be missing church or negative impact of the pandemic on their spiritual practices. *“I am most concerned about not being able to worship collectively with my church.”*
Concerns for other	Expresses any concern about others other than themselves. Includes both known and general public. (Note: As illustrated in the quotes below, concerns could reflect opinions about the behavior of others as well as concern for the well-being of others). *“I live in a small courtyard and three of my neighbors spend hours and hours in each other’s bungalow talking and have given up on social distancing and came up with movie night in which they share popcorn and snacks – that is people putting their hands in a bowl and grabbing snacks/popcorn. They seem to think that that since they stay home it’s okay if they spend hours talking to each other, but one goes out and works and the other two sometimes do go out for whatever reason.”* *“That things will get back to normal and the world can start being healthy again, too many people are suffering from this virus. A lot of people are also being persecuted economically which is devastating.”*

Ninety-two percent (*n* = 461) of participants indicated at least one concern that was categorized per the coding scheme, with *M* = 1.3*, SD* = 0.72 (see Table 3). We observed how frequently each type of concern was indicated across participants (see Table 4). The most common concerns were Concern for Others (*n* = 142, 28%), Physical Health (*n* = 136, 27%), and Social (*n* = 120, 24%). The majority of participants (*n* = 352, 70%) endorsed a high degree of concern severity (scores of 4–5 out of 5), with an average rating of 4.03 (*SD* = 1.04) about the concern(s) mentioned in their open-ended response.

**Table 3:** Frequency of the total number of types of concerns observed in participant responses to the open-ended pandemic-related concerns question (*N* = 501).

Total number of types of concerns observed	*n*	%
0	40	8.0
1	301	60.1
2	135	26.9
3	20	4.0
4	4	0.8
5	1	0.2

**Table 4:** Frequency of the types of concerns observed in participant responses to the open-ended pandemic-related concerns question (*n* = 501).

	*n*	%
Concerns for others	142	28.3
Physical Health	136	27.1
Socializing	120	24.0
Work/Finance/Employment	74	14.8
Socio-Political- Economic	70	14.0
Psychological Health and Mental Wellness	33	6.6
Mask Wearing	30	6.0
Access to Food	24	4.8
Access to Hygienic or Household Supplies	15	3.0
Spiritual	6	1.2
Access to Services	2	0.4

### 3.3. Pandemic-related consequences checklist

Ninety-nine percent (*n* = 495) of our sample endorsed one or more pandemic related consequence on the 10-item checklist developed by the research team (*M* = 3.70, *SD =* 1.93; see Table 5). The most common consequences were related to social activities, with 83% (*n* = 417) reporting at least one of the three social activities items: not attending social events or group activities in the greater community, not attending social events or group activities at your own residence, and not seeing family. The next most common consequences were related to physical health, with 45% (*n* = 226) endorsing not getting physical exercise and/or disruption in needed medical care. The least common consequence was not going outside (*n* = 111, 22%).

**Table 5:** Frequency of participants experiencing each of 10 potential pandemic-related consequences (*N* = 501).

	*n*	%
Not attending social events or group activities in the greater community	287	57.3
Haven’t seen family	279	55.7
Not running usual errands	278	55.5
Not attending social events or group activities at your residence	231	46.1
Concern over finances	204	40.7
Not getting physical exercise	151	30.1
Difficulties acquiring groceries or other essentials	142	28.3
Disruption in needed medical care	126	25.1
Not going outside	111	22.2
Other consequence	45	9.0
No consequence checked	6	1.2

### 3.4. Comparison of responses to the open-ended pandemic-related concerns question and the pandemic-related consequences checklist items

There was overlap between the types of experiences participants indicated having on the consequences checklist items as well as the open-ended concerns question; however, there were also unique concerns identified that were not captured by the checklist items. Specifically, with regards to social activities, we observed an additional 1% (*n* = 7) of participants who mentioned a concern that was coded as Socializing based on their response to the open-ended pandemic-related concerns question but who did not report a social activity consequence on the checklist. Additionally, although 83% of participants reported a social activity consequence on the checklist, only a subset (*n* = 120, 24%) mentioned a concern that was coded as Socializing. Overall, 85% (*n* = 424) of participants endorsed either a social consequence or concern.

With regards to physical health, we observed an additional 11% (*n* = 57) of participants with a Physical Health concern on the open-ended pandemic-related concerns question that did not report a physical health consequence on the checklist. Additionally, although 45% (*n* = 226) of participants reported a physical health consequence, only a subset (*n* = 136, 27%) indicated a Physical Health pandemic-related concern. Overall, 57% (*n* = 283) of participants endorsed either a physical health consequence or concern.

With regards to finance, we observed an additional 2% (*n* = 12) of participants with a Work/Finance/Employment concern on the open-ended pandemic-related concerns question that did not report a financial consequence on the checklist. Additionally, although 40% (*n* = 204) of participants reported a financial consequence, only a subset (*n* = 74, 15%) indicated a Work/Finance/Employment pandemic-related concern. Overall, 43% (*n* = 216) of participants endorsed either a financial consequence or concern.

With regards to access to food, we observed 2% (*n* = 8) of participants with an Access to Food concern on the open-ended pandemic-related concerns question that did not report an access to difficulties acquiring groceries or other essential consequence on the checklist. Additionally, although 28% (*n* = 142) of participants reported an access to groceries or other essentials on the checklist, only a subset indicated an Access to Food concern 5% (*n* = 24). Overall, 30% (*n* = 150) of participants endorsed either an access to food or other supplies consequence or concern.

## 4. Discussion and implications

Three months into the global COVID-19 pandemic, the types of concerns older adults mentioned in the open-ended pandemic-related concerns question and the types of consequences they reported experiencing up to that point in the pandemic are consistent with what might be expected following social distancing policies that discouraged social interaction outside of the home and resulted in closed business and reduced in-person services, such as social interaction and activity, physical health, and not being able to run their usual errands. These types of disruptions are concerning for the well-being of older adults, as staying physically and socially active are essential components of healthy aging (Rowe and Kahn, 1997).

We observed evidence of an impact on finances as well, with 43% of older adults expressing either a concern on the open-ended question related to finances or having experienced a negative financial consequence because of the pandemic on the checklist items. Importantly, a considerable proportion of older adults in our sample reported that they either had “just enough to get by” or “cannot make ends meet” (58%). Thirteen percent of older adults live in poverty, as defined by the Supplemental Poverty Measure (Li and Dalaker, 2021), which is similar to the proportion (10%) of our sample reported not having enough money to “make ends meet.” The impact of the pandemic and pandemic-related policies on older adults’ financial well-being could be detrimental as socio-economic status and psychological distress are highly associated (Ross and Mirowsky, 2006; Spence et al., 2011).

We observed evidence of an impact of COVD-19 on access to food and other essentials such that 30% of older adults expressed either a concern on the open-ended question or having experienced a negative consequence related to this on the checklist items. We were not able to ascertain whether these responses reflected inconveniences, such as not being able to find preferred brands, or if they reflected circumstances that are severe, such as going hungry due to restricted access to food sources. Prior to the pandemic, it was reported that 7% of seniors were food insecure in 2019 (Ziliak and Gundersen, 2019) and according to Conservation of Resources Theory (Hobfoll and Ford, 2007), psychological stress occurs when resources are threatened or lost in instances like the COVID-19 pandemic. Future studies could ask specifically about food scarcity and limitations in the supply chain for critical food items.

The data collection period (June of 2020) coincides with the killing of George Floyd and the protests, civil unrest, and increased awareness of police brutality that occurred following his death. Concerns about this topic were coded as socio-political-economic concerns. While we did not commonly observe this concern in participants’ responses to the concern question (*n* = 70, 14%), it is possible it was under-reported as the question was framed specifically around the pandemic and its consequences. A question specifically about social justice and racial equity may have resulted in more of these concerns. For example, Igarashi and colleagues found socio-political-economics themes in cultural divide (20%) and concern for society (9%; Igarashi et al., 2022).

A minority of participants mentioned a concern that was coded as Psychological Health and Mental Wellness on the open-ended concerns question (7%). Our team previously published findings that 24% of participants in this sample reported psychological distress based on measures of depression and anxiety (Sams et al., 2021). The relatively low frequency of older adults mentioning psychological concerns - both in comparison to the frequency of other types of concerns and in light of the rate of distress observed in this sample in previous work - may reflect the fact that although older adults were experiencing psychological distress 3 months into the pandemic, it may not have been a primary concern for them at that point. In fact, a similar phenomenon has been observed in patients experiencing early symptoms of posttraumatic stress after a traumatic injury event, which is the context from which our open-ended concerns question originated (Zatzick et al., 2001). For instance, despite being enrolled in a clinical trial based on experiencing clinically significant symptoms of posttraumatic stress early after trauma, patients enrolled in the trial more frequently reported physical and work/financial concerns than psychological concerns in the months following a serious injury (Zatzick et al., 2018). Whether older adults perceive psychological distress symptoms as a concern could influence how readily they seek support or mental health care for these symptoms.

Other concerns categories that were reported in low frequency, including Mask Wearing (6%), Spiritual, such as missing church services (1%), and Access to Services (<1%). Interestingly, only a small percentage of our respondents shared a concern that had any mention of masks. The low rates in Spiritual and Access to Services concerns could reflect the large-sweeping adoption of technological support such as video-conference technology to boost access to services and spiritual practices.

An important distinction our data illustrate is the difference between experiencing a disruption to life activities as a consequence of a pandemic and perceiving that consequence to be of great personal concern. This is highlighted by our data showing that, for instance, although participants frequently reported reduction in social activity on the consequences checklist items, only a subset of those participants mentioned Socializing as a concern on the open-ended concerns question. This may reflect variability in psychological reactions to the same circumstances such that what is perceived as stressful by one person may not be to another, or could perhaps even be perceived as a benefit. For instance, it could be that some participants actually found the reduction in social activities to be a positive shift in their life. Both the experience of a stressor and person’s perception of that stressor are meaningful to mental health outcomes (Keller et al., 2012). A thorough approach to inquiry about the impact of the pandemic over time might include both what older adults have experienced as consequences of the pandemic as well as their personal reaction to these experiences.

### 4.1. Comparison with previous qualitative studies of older adults’ pandemic-related concerns

Methodological differences make it challenging to compare findings from our study with those of previous survey studies using qualitative data to explore older adults’ experiences. First, three previous qualitative studies asked older adults about stressors and difficulties experienced earlier in the pandemic than our study; one just after social distancing policies went into effect and the other between 1 and 2 months later. The timing of data collection may have impacted findings across these studies. For instance, personal physical health concerns were infrequent in Whitehead and colleagues study (Whitehead and Torossian, 2020) and Heid and colleagues study (Heid et al., 2021) and were not an observed theme in that of Igarashi and colleagues (Igarashi et al., 2022); however, this was one of the most frequent concerns mentioned in our study. It may be that as more time passed, the experience of contracting or witnessing others contracting the virus may have increased, resulting in greater concern about this. We may have observed more financial concerns in our sample than participants in these studies for a similar reason. Alternatively, the low reports of financial concerns in previous studies could be related to socioeconomic and demographic differences. For instance, our sample is more racially and ethnically diverse than the other studies. Whitehead et al.’s and Heid et al.’s samples had higher income than ours and income was not reported in the Igarashi et al. paper.

Another methodological difference is that the Whitehead et al. and Igarashi et al. studies asked about stressors/difficulties occurring at the time of data collection, whereas our study and Heid et al.’s inquired about concerns or challenges since the start of the pandemic. Immediate concerns could be different than what has been a concern over time. For instance, a participant who lost their job may be overall quite concerned about finances and employment, but if they contract COVID-19, at that period of time they may be most concerned about their physical health. Asking the question in both ways could help us understand the chronic and immediate concerns associated with pandemic.

A final methodological difference is that previous studies and ours applied idiosyncratic coding schemes to open-ended responses. Although some similar themes were identified, the operational definitions varied, which would impact frequencies observed for each theme. For instance, our theme of Concerns for Others was broadly inclusive and likely encompassed stressors reflected in both Whitehead et al.’s Concern for Others theme as well as “Other People” (concern for how other people were responding to the pandemic), which may explain why our rates were higher for Concern for Others than theirs. Similarly, Igarashi et al.’s theme of Psychological Distress appears to be broader than ours of Psychological Health and Mental Wellness, encompassing concerns such as boredom and uncertainty about the future, which may explain why their rates were higher than ours. Heid et al.’s category of psychological stressors includes worries that we incorporated in other concern categories; for instance, they coded worries about other people as a psychological stressor whereas we coded this under Concern for Others. As research in this area progresses, common themes and operational definitions may emerge that could be applied consistently across samples to gain a clearer picture.

### 4.2. Limitations and considerations

There are methodologic limitations of the study to consider. With regard to generalizability, our sample taken from online platforms of MTurk and Prolific and may reflect older adults who are generally healthy, technologically active, and well-educated (Turner et al., 2020). Findings also represent experiences of older adults based at one time-point in an unfolding pandemic; however, experiences and concerns of older adults may change over the course of the pandemic. The order of survey questions could have influenced the findings. Specifically, the consequences checklist was administered before the open-ended pandemic concerns question, which may have primed participants to mention concerns similar to the items on the consequence checklist versus illuminating novel concerns. Despite this; however, the concerns question did elicit novel responses that were not previously included in the consequence checklist of items. Although the open-ended concerns question illustrated the diversity of concerns older adults have in a pandemic, the question “what concerns you the most” may have restricted the range of concerns reported by each participant. Most participants (60%) indicated only one concern; however, indicating only one concern does not mean that older adults did not have other concerns, and future survey research could aim to elicit all of the concerns people have.

Our data speak to the causal impact of the pandemic insofar as participants responded to questions that inquired specifically about consequences to engaging in various activities because of the pandemic on the consequences checklist. Our qualitative data could speak to both concerns caused by the pandemic or other co-occurring events, such as the increased awareness of racial discrimination as previously noted. Future use of the open-ended concerns question could restate the question to read, “Of all the things that have happened to you *because* of the COVID-19 pandemic started, what concerns you the most?” Although such wording may be useful for making stronger causal statements, a survey designed to identify the needs of a population in the context of a pandemic may make use of the broader question so as not to miss experiences that may be important to address from a public health perspective.

Finally, although this study reflects a nationally representative sample for racial identities, there was inadequate sample size for subgroup analyses by race/ethnicity. It is important to continue to aim for both racially representative samples and sample sizes that allow the exploration of between-group differences, especially when the COVID-19 pandemic is known to differentially affect racial/ethnic groups (Bui et al., 2021; Garcia et al., 2021).

### 4.3. Implications for the psychology of aging and public health

A common type of concern mentioned in the open-ended question was Concerns for Others (28%). This code covers both concerns for others that they know, such as friends and family, as well as people they did not know and spans both concerns about others’ well-being and their risky behaviors. Our findings along with those of previous studies suggest older adults are concerned about the interpersonal consequences of the pandemic as well as the well-being of other people or society at-large. These findings align well with the Socioemotional Selectivity Theory (Carstensen et al., 1999). According to this theory, age is associated with a sense (perceived consciously or not) that the amount of time left to live is limited, resulting in a shift in motivation for socioemotional experiences toward those that are most meaningful in present time (e.g., time spent with close friends) and away from experiences that are novel and future-oriented (e.g., engaging in activities to meet new people). Related, older adults are often more motivated to pursue prosocial goals (Carstensen and Chi, 2021). Therefore, efforts to support older adults in engaging in meaningful social connection despite the requirement for social distancing is an important public health strategy. Many such programs include education regarding the awareness of social isolation and tips about how to reach out to others, be physically active and use technology as a means of staying connected (Administration for Community Living, 2020b; NAMICA, 2022).

The use of technology has played a key role for in sustaining social connection during the pandemic, with 2021 Pew Research estimates of 81% of Americans using video calling and conferencing during the pandemic (Mcclain et al., 2021); however, older adults are known to have more barriers to accessing and utilizing technology than other age groups (Litchfield et al., 2021) and less frequently reported that using technology during the pandemic was essential relative to other adults (Mcclain et al., 2021). Interventions to support technology literacy and increase access to diverse types of technologies (e.g., email, video calls, social media, virtual gathering spaces) for meaningful social engagement are important considerations for supporting older adults in the context of potential future social distancing policies associated with future pandemics (O’Connell et al., 2022).

Knowing what older adults are concerned about and motivates them can help focus public health messaging, which is particularly salient in the context of a health crisis like the COVID-19 pandemic. For instance, given that older adults are concerned about the well-being of others, effective public health messaging could help them attend to the needs and well-being of loved ones as well as their own. An example would be the campaign “I Wear a Mask Because” as part of the COVID-19 Public Education Campaign, where people share their concerns regarding the spread of COVID-19, in hopes to motivate others to mask up (U.S. Department of Health and Human Services, 2020). Such messaging is also consistent with the Socioemotional Selectivity Theory and the resulting guidance based on this theory to promote effective public health messaging (Carstensen and Hershfield, 2021). For instance, messages that focus on the meaningful rewards gained by a health behavior (e.g., “vaccinations help us keep others safe”) as well as the positive versus negative implications of health behaviors (e.g., “vaccinations help us return to usual activities”) are likely to be most effective for older adult populations.

We were able to assess what concerns older adults had during a pandemic using a brief open-ended question. Asking for people’s concerns using an open-ended question allows them to share their experience in a person-centered way and places value on understanding each person’s unique experience. As modeled in foundational research in which patients are asked about concerns after a serious injury (Zatzick et al., 2001), addressing patients most pressing concerns during a stressful experience can be an effective strategy for engaging people in preventive services that may ultimately be helpful in addressing psychological distress should that arise and is consistent with best-practices for supporting psychological recovery for people after a disaster or other trauma (Zatzick et al., 2018; Wang et al., 2021; O’Connell et al., 2022). Given that we were able to gather such rich data via a web-based survey, it may be that routine administration of the concerns question utilized in this study could be integrated across types of survey administration (e.g., phone, mail, interview, or web-based) and could be used to monitor the types of concerns older adults have at a population-level as well as be useful in guiding specific supportive interventions for individual older adults.

## 5. Conclusion

Although older adults endorsed a variety of social, physical health, and financial/ economic consequences of the COVID-19 pandemic 3 months into the pandemic, their self-reported pandemic-related concerns most frequently reflected concern about the well-being and behavior of others, one’s own physical health, and the impacts of the pandemic and social distancing policies on social activities. Consistent with these findings and the Socioemotional Selectivity Theory, it may be particularly important to support older adults during a pandemic by facilitating meaningful social engagement and encouraging health behaviors through prosocial and positive public health messaging.

## Data availability statement

The raw data supporting the conclusions of this article will be made available by the authors, without undue reservation.

## Ethics statement

The studies involving humans were approved by University of Washington Human Subjects Division. The studies were conducted in accordance with the local legislation and institutional requirements. The ethics committee/institutional review board waived the requirement of written informed consent for participation from the participants or the participants’ legal guardians/next of kin because the informed consent process was conducted via an online survey.

## Author contributions

NS, DD, BM, and PA contributed to the conception and design of the study. NS, DF, RA, and KH contributed to the coding and qualitative analysis. NS and DD contributed to the statistical analysis and first drafts of manuscript. All authors contributed to the manuscript revision, read, and approved the submitted version.
